# Supplementary material for: Chromothripsis during telomere crisis is independent of NHEJ, and consistent with a replicative origin
Source: Genome Res. 2019 May;29(5):737–49. doi: 10.1101/gr.240705.118 (PMC6499312; doi:10.1101/gr.240705.118)
Supplement: Supplemental Material [file supp_gr.240705.118_Supplemental_file_1.zip › contigs/annotated_contigs/DB37/contig.2.DB37_length_378_mean_cov_4.36507936508.docx]

**DB37_length_378_mean_cov_4.36507936508**

```
GGAAGTTCATACAAGCTCTCTCATTTAATTATCTCTCCAATCCTGAGGGGTATTAAAAACTCTTATTATATAGTTGAAGTTATTGAGAG
 >chr9:22530998-22531151 + E=1e-80
AAGGTCTTGCATTTAGTAAGTCACCAAGTAGTCATTGAACCTTGGTTCTCTTTTATTCTAAA|GT|GTGCTGCACCCATTAACTCGTCA
                                                               >chr9:22531650-22531871
TTTACATTAGGTATATCTCCTAATGCTATCCCTCCCTCCTCCCCCACCCCACAACAGGCCCTGGTGTGTGATGTTCCCCTTCCTGTGTC
+ E=1e-103
CAAGTGTTCTCATTGTTCAATTCCCACCTGTGAGTGAGAACATGCGGTGTTTGGTTTTTTGTCCTTGCGCTAGTATTGCTGTTATGACG

ATTCATGCACATGTATGT|ATATGC
```
